# Development and Delphi Validation of a Comprehensive High Reliability Program to Improve Nursing Work Environment and Patient Safety

**DOI:** 10.7759/cureus.107677

**Published:** 2026-04-24

**Authors:** Abdulaziz S Almutairi, Faridah Mohd Said

**Affiliations:** 1 Nursing, Lincoln University College, Petaling Jaya, MYS

**Keywords:** delphi technique, healthcare quality improvement, high reliability organization, nursing work environment, patient safety

## Abstract

Background: Patient safety remains a critical global healthcare priority, with the nursing work environment recognized as a key determinant of safety outcomes. While existing studies have established associations between work environment factors and patient safety, there is a lack of structured, evidence-based interventions that translate these findings into practical frameworks. This study aimed to develop and validate a comprehensive High Reliability Program (HRP) designed to improve the nursing work environment and enhance patient safety.

Methods: A methodological study design employing a modified Delphi technique was used. The HRP was developed based on empirical literature and theoretical frameworks, including the Job Demands-Resources model and Systems Theory. The program comprised five core domains: orientation, guidance and mentorship, continuous support, feedback and evaluation, and integration into the work environment. A purposive sample of 10 experts in nursing, patient safety, and healthcare management participated in three Delphi rounds. Content validity was assessed using the Content Validity Index (CVI) and Content Validity Ratio (CVR), with a predefined threshold of ≥0.80.

Results: The Delphi process achieved a 100% response rate across all rounds. Initial feedback led to the refinement of program components to enhance clarity, feasibility, and clinical applicability. Progressive agreement was observed across rounds, culminating in full consensus by the third round. All items achieved maximum validity scores (CVI = 1.00; CVR = 1.00), indicating unanimous agreement on their relevance and essentiality. The final HRP was structured as a comprehensive, modular intervention addressing key determinants of the nursing work environment, including communication, leadership, training, and organizational support.

Conclusions: The study successfully developed and validated a comprehensive HRP with excellent content validity. The HRP provides a structured, evidence-based framework for improving the nursing work environment and strengthening patient safety culture. Its modular and adaptable design supports implementation across diverse healthcare settings, particularly in resource-constrained environments. Future research should evaluate the effectiveness of the HRP through implementation studies and outcome-based assessments.

## Introduction

Patient safety remains a fundamental priority in contemporary healthcare systems, reflecting the commitment to minimizing harm and ensuring the delivery of high-quality care. Despite substantial advancements in medical technology and clinical practice, preventable adverse events continue to pose a significant global health burden. Reports have consistently highlighted that medical errors contribute to considerable morbidity and mortality, with earlier estimates indicating that tens of thousands of deaths annually are attributable to preventable incidents within healthcare settings [1,2]. More recent evidence suggests that unsafe care not only compromises patient outcomes but also increases healthcare costs and undermines public confidence in health systems [3]. Consequently, improving patient safety has become a central objective for healthcare organizations worldwide.

Within this context, the role of nurses is particularly critical, as they operate at the frontline of patient care and are directly involved in monitoring, treatment administration, and clinical decision-making. The quality of care delivered by nurses is strongly influenced by the environment in which they work. The nursing work environment encompasses a complex interplay of organizational, structural, and interpersonal factors, including staffing levels, leadership support, communication patterns, workload, and access to resources [4,5]. Evidence consistently demonstrates that a supportive and well-resourced work environment enables nurses to perform effectively, adhere to safety protocols, and engage in collaborative practice, thereby enhancing patient safety outcomes [6]. Conversely, unfavorable work conditions, such as staff shortages, excessive workload, and poor communication, are associated with increased risks of adverse events, including medication errors, infections, and patient falls [7,8].

The relationship between the nursing work environment and patient safety is further influenced by organizational culture and leadership. A positive safety culture, characterized by open communication, teamwork, and nonpunitive responses to error reporting, fosters an environment in which nurses feel empowered to identify and address potential risks [9]. In contrast, environments marked by hierarchical barriers and ineffective communication can suppress reporting behaviors and contribute to preventable harm [10,11]. Leadership plays a pivotal role in shaping these dynamics, as supportive and transformational leadership styles have been shown to promote safety culture, enhance job satisfaction, and improve patient outcomes [12,13].

In response to these challenges, the concept of high-reliability organizations (HROs) has gained increasing attention in healthcare. HROs are characterized by their ability to operate in complex, high-risk environments while maintaining consistently low error rates. These organizations emphasize principles such as preoccupation with failure, sensitivity to operations, and commitment to resilience, all of which are essential for improving patient safety. Translating these principles into structured interventions within healthcare settings has the potential to address both organizational and clinical determinants of safety. However, despite growing recognition of the importance of high-reliability principles, there remains a limited number of comprehensive, evidence-based programs specifically designed to integrate these principles into nursing practice and the broader work environment.

A critical gap in the existing literature lies in the lack of structured, validated programs that simultaneously target the nursing work environment and patient safety. While numerous studies have explored the relationship between these variables, most have focused on observational or correlational analyses rather than the development of actionable interventions [8,14]. Furthermore, the majority of existing research has been conducted in large tertiary hospitals, with limited attention given to secondary healthcare settings that often face unique challenges, including resource constraints, staffing limitations, and reduced access to specialized training programs [15,16]. This gap underscores the need for context-specific, sustainable interventions that can be implemented effectively across diverse healthcare environments.

To address this limitation, the present study focuses on the development of a comprehensive High Reliability Program (HRP) tailored to improve the nursing work environment and enhance patient safety. Given the complexity of healthcare systems and the multifaceted nature of patient safety, it is essential that such programs are rigorously validated prior to implementation. The Delphi technique, a structured method for achieving expert consensus, is widely recognized as a robust approach for establishing content validity in healthcare research. By systematically incorporating expert opinions, the Delphi method ensures that the components of an intervention are relevant, comprehensive, and aligned with current best practices.

Therefore, the aim of this study was to develop a comprehensive HRP based on empirical evidence and established theoretical frameworks and to establish its content validity using a modified Delphi technique. By providing a validated, evidence-based framework, this study seeks to contribute to the advancement of nursing practice and patient safety while offering a practical tool for healthcare organizations striving to achieve high reliability in clinical care.

## Materials and methods

Study design

This study employed a methodological research design utilizing a modified Delphi technique to develop and validate a comprehensive HRP aimed at improving the nursing work environment and patient safety. The study was conducted in two sequential phases: (1) development of the HRP based on empirical evidence and theoretical frameworks, and (2) validation of the program through expert consensus using the Delphi method. This approach is widely recognized for establishing content validity in healthcare interventions, particularly when empirical evidence requires supplementation with expert judgment.

Phase I: development of the HRP

The initial development of the HRP was informed by a comprehensive review of the literature on the nursing work environment and patient safety, alongside established theoretical frameworks, including the Job Demands-Resources (JD-R) model and Systems Theory. A narrative synthesis approach was employed to extract key determinants-such as communication, leadership, training, organizational support, and professional development-which were subsequently grouped into thematic categories. These themes were iteratively refined and aligned with the underlying theoretical constructs, resulting in the development of five core domains: orientation, guidance and mentorship, continuous support, feedback and evaluation, and integration into the work environment. The HRP was designed as a structured, evidence-based intervention comprising five core domains (Table 1).

**Table 1:** Components of a Comprehensive High Reliability Program for Enhancing Nursing Work Environment and Patient Safety

Step	Description
Orientation	Introduction of nurses to safety principles, organizational expectations, and clinical protocols
Guidance and mentorship	Structured support through experienced staff to enhance clinical competence and teamwork
Continuous support	Ongoing professional development and reinforcement of safety practices
Feedback and evaluation	Systematic performance assessment and constructive feedback mechanisms
Integration into the work environment	Embedding learned behaviors and safety practices into routine clinical workflows

These domains were developed to address key determinants of the nursing work environment, including leadership, communication, training, and organizational support, which have been shown to influence patient safety outcomes [7,14]. The preliminary version of the HRP was organized into structured modules and prepared for expert evaluation.

Phase II: Delphi validation of the HRP

Expert Panel Selection

A purposive sampling approach was used to recruit experts with relevant clinical, academic, and managerial expertise in patient safety and healthcare quality improvement. Participants were identified through professional networks, academic affiliations, and clinical leadership roles. Inclusion criteria required a minimum of a bachelor’s degree in nursing or a related healthcare field, at least five years of professional experience, and demonstrated expertise in patient safety, nursing leadership, or quality improvement.

The panel was composed to ensure diversity across professional roles, including clinical practice, nursing education, and healthcare management, and included participants from both clinical and academic institutions. While efforts were made to enhance diversity of perspectives, geographic representation was limited to experts accessible within established professional networks.

Delphi Procedure

The Delphi process was conducted over multiple rounds to achieve consensus on the content and structure of the HRP. In the first round, experts were provided with the preliminary HRP modules and asked to evaluate each component in terms of relevance, clarity, and applicability. They were also encouraged to provide qualitative feedback and suggestions for improvement.

Subsequent rounds incorporated revisions based on expert feedback, allowing participants to reassess the modified content. This iterative process continued until a satisfactory level of agreement was achieved. The anonymity of responses was maintained throughout the process to minimize bias and ensure independent judgment.

Data Collection Instrument

A structured evaluation questionnaire was developed to facilitate expert assessment of the HRP components. Each item was rated using a four-point Likert scale ranging from “not relevant” to “highly relevant.” The use of a four-point scale eliminated neutral responses and encouraged decisive evaluation of each component.

Validity Measures

Content validity was assessed using the Content Validity Index (CVI) and Content Validity Ratio (CVR). The CVI was calculated as the proportion of experts rating each item as relevant or highly relevant, while the CVR was computed to determine the essentiality of each item based on expert judgment.

A predefined threshold of ≥0.80 was considered acceptable for item retention, consistent with established methodological standards. Items that did not meet this threshold were revised or removed based on expert feedback.

Data Analysis

Descriptive statistics were used to summarize expert ratings and demographic characteristics. CVI and CVR values were calculated for each item and for the overall scale to determine the level of consensus. Changes across Delphi rounds were analyzed to assess the progression toward agreement. All analyses were conducted using standard statistical procedures.

Ethical considerations

Ethical approval for the study was obtained from the relevant institutional review authority. Participation in the Delphi process was voluntary, and informed consent was obtained from all experts prior to data collection. Confidentiality and anonymity were strictly maintained throughout the study, and all data were used solely for research purposes.

This methodological approach ensured the systematic development and rigorous validation of the HRP, providing a robust foundation for its subsequent implementation and evaluation in clinical settings.

## Results

Characteristics of the expert panel

A total of 10 experts participated in the Delphi validation process. The panel comprised professionals with diverse expertise in clinical nursing, nursing education, and healthcare management. All participants met the predefined inclusion criteria, possessing substantial experience in patient safety, quality improvement, and nursing leadership.

The expert panel demonstrated a high level of academic and professional competence, with the majority holding postgraduate qualifications and having more than five years of experience. This ensured that the validation process was informed by both theoretical knowledge and practical clinical insight. The detailed demographic and professional characteristics of the expert panel are presented in Table 2.

**Table 2:** Characteristics of the Expert Panel

Demographic		Frequency	Percentage
Gender	Male	5	50
	Female	5	50
Experience	5-7 years	2	20
	8-10 years	2	20
	More than 10 years	6	60
Expertise Role	Nurse Educators	2	20
	Patient Safety Coordinator / Specialist	2	20
	Nursing Managers	2	20
	Clinical Nurse Specialists	2	20
	Nursing Quality / Improvement Coordinator	2	20

Evidence-based development of the HRP

Prior to the Delphi validation process, the comprehensive HRP was developed based on empirical literature and theoretical frameworks, including the Job Demands-Resources model and Systems Theory. Each component of the program was systematically mapped to evidence-based practices to ensure relevance to both the nursing work environment and patient safety. The mapping of HRP domains and components to supporting evidence is presented in Table 3.

**Table 3:** Evidence-Based Mapping of HRP Modules and Components HRP: High Reliability Program.

Module	Component	Supporting Literature	Evidence-Based Contribution
Orientation Module	Organizational overview and policies	Pramesona et al. [17] (2023)	Improves organizational integration and safety culture awareness
	Role expectations and responsibilities	Yoo and Kim [6] (2018)	Enhances role clarity and reduces clinical errors
	Patient safety fundamentals	Ulrich et al. [5] (2022)	Strengthens adherence to safety protocols
Guidance and Mentorship Module	Mentorship and professional support	Montgomery et al. [4] (2022)	Improves clinical competence and confidence
	Communication in handoffs	Mahmoud et al. [9] (2023)	Reduces communication-related errors
	Incident reporting systems	Kakemam et al. [10] (2020)	Promotes error reporting and safety culture
Continuous Support Module	Medication safety training	Malinowska-Lipień et al. [14] (2021)	Reduces medication errors
	Infection prevention and control	Lee et al. [7] (2023)	Reduces hospital-acquired infections
	Risk identification and prevention	Lee et al. [7] (2023)	Enhances proactive risk management
	Stress management strategies	Moisoglou et al. [18] (2020)	Mitigates workload-related performance decline
Feedback and Evaluation Module	Performance evaluation systems	Ulrich et al. [5] (2022)	Enhances accountability and performance monitoring
	Feedback mechanisms	Kakemam et al. [10] (2020)	Supports continuous improvement
Integration into Work Environment Module	Professional development	Vlasblom et al. [19] (2020)	Sustains long-term competency and safety practices
	Teamwork and collaboration	Tamata and Mohammad [20] (2023)	Improves interdisciplinary coordination
	Leadership development	Ulrich et al. [5] (2022)	Strengthens organizational support and safety culture

Round 1 Delphi results

In the first Delphi round, the preliminary HRP was evaluated by the expert panel. Overall, the results demonstrated a high level of agreement across most items, indicating that the program components were generally relevant and appropriate.

However, several items required refinement based on expert feedback. The suggested modifications focused on improving clarity, enhancing alignment with clinical practice, and ensuring feasibility within routine healthcare settings. Experts also emphasized the importance of integrating practical considerations, particularly in relation to communication strategies and mentorship structures. The detailed results of round 1, including item-level agreement and validity indices, are presented in Table 4.

**Table 4:** Round 1 Results PAS: Percentage Agreement Score.

Module Number	Module Title	Session Title	Mean ± SD	Median (IQR)	PAS
Day 1	General Orientation	Welcome Session: Overview of the Organization	4.50 ± 0.53	4.50 (1.00)	90%
		Facility Tour and Team Introduction	4.30 ± 0.48	4.00 (1.00)	86%
		Organizational Policies and Culture	4.50 ± 0.53	4.50 (1.00)	90%
Module 1	Orientation	Health and Safety Training (Evidence-Based)	4.70 ± 0.48	5.00 (1.00)	94%
		Role Expectations and Responsibilities	4.70 ± 0.48	5.00 (1.00)	94%
		Patient Safety Essentials: Understanding Risk Factors	4.70 ± 0.48	5.00 (1.00)	94%
		Assigning a Mentor and Building Professional Relationships	4.40 ± 0.52	4.00 (1.00)	88%
Module 2	Guidance and Mentorship	Setting Professional Goals With the Mentor	4.70 ± 0.48	5.00 (1.00)	94%
		Introduction to Key Team Members	4.70 ± 0.48	5.00 (1.00)	94%
		Safe Communication in Patient Handoffs	4.50 ± 0.53	4.50 (1.00)	90%
		Introduction to Incident Reporting Systems	4.40 ± 0.52	4.00 (1.00)	88%
		Operational Challenges and Evidence-Based Solutions	4.80 ± 0.42	5.00 (0.25)	96%
		Access to Resources and Tools (Evidence-Based)	4.60 ± 0.52	5.00 (1.00)	92%
Module 3	Continuous Support	Stress Management Strategies	4.40 ± 0.52	4.00 (1.00)	88%
		Identifying and Preventing Patient Safety Risks	4.60 ± 0.52	5.00 (1.00)	92%
		Medication Safety and Administration	4.60 ± 0.52	5.00 (1.00)	92%
		Infection Prevention and Control	4.60 ± 0.52	5.00 (1.00)	92%
		Monthly Continuing Education Sessions	4.70 ± 0.48	5.00 (1.00)	94%
Module 4	Feedback and Evaluation	Performance Evaluations and Goal Adjustment (Evidence-Based)	4.50 ± 0.53	4.50 (1.00)	90%
		Collecting Employee Feedback (Surveys and Interviews)	4.70 ± 0.48	5.00 (1.00)	94%
		Applying Feedback to Practice	4.70 ± 0.48	5.00 (1.00)	94%
		Team-Building Activities and Cohesion	4.40 ± 0.52	4.00 (1.00)	88%
		Leadership and Professional Growth Opportunities	4.40 ± 0.52	4.00 (1.00)	88%
Module 5	Integration Into Work Environment	Professional Development Workshops	4.50 ± 0.53	4.50 (1.00)	90%
		Long-Term Career Path Planning	4.60 ± 0.52	5.00 (1.00)	92%
		Improving Patient Safety Outcomes Through Collaboration	4.40 ± 0.97	5.00 (1.00)	88%
Added Session Contents From Open-ended Questions	1. The Art of Receiving and Applying Feedback 40% 2. Understanding team Dynamics and collaboration 50% 3. Navigating ethical dilemmas in daily practice 50% 4. Advanced communication strategies for patient advocacy 50%			Response rate = 100%	

Round 2 and round 3 results

Following revisions to the HRP, subsequent Delphi rounds were conducted to reassess the modified program. The results demonstrated a progressive increase in agreement among experts, reflecting the effectiveness of the revisions.

In round 2, most items achieved high levels of agreement, with only minor adjustments required. These refinements were primarily related to wording, sequencing, and overall coherence of the program structure.

By Round Three, full consensus was achieved across all items. All components were rated as clear, relevant, and essential, indicating stability in expert responses and completion of the Delphi process. The detailed findings from rounds 2 and 3 are presented in Table 5.

**Table 5:** Rounds 2 and 3 *Added. PAS: Percentage Agreement Score; IQR: Interquartile Range; CVR: Content Validity Ratio; CVI: Content Validity Index.

Module Number	Module Title	Session Title	Round 2					Round 3				
			Median (IQR)	Mean ± SD	% PAS	CVR Score	CVI Score	Median (IQR)	Mean ± SD	% Agreement	CVR Score	CVI Score
Day 1	General Orientation	Welcome Session: Overview of the Organization	5.00 (0.00)	4.82 ± 0.42	96.40%	1	1	5 (0.00)	5.00 ± 0.00	100%	1	1
		Facility Tour & Team Introduction	5.00 (0.00)	4.91 ± 0.32	98.20%	1	1	5 (0.00)	5.00 ± 0.00	100%	1	1
		Organizational Policies & Culture	5.00 (1.00)	4.73 ± 0.48	94.60%	1	1	5 (0.00)	5.00 ± 0.00	100%	1	1
Module 1	Orientation	Health & Safety Training (Evidence-Based)	5.00 (0.00)	4.82 ± 0.42	96.40%	1	1	5 (0.00)	5.00 ± 0.00	100%	1	1
		Role Expectations & Responsibilities	5.00 (1.00)	4.73 ± 0.48	94.60%	1	1	5 (0.00)	5.00 ± 0.00	100%	1	1
		Patient Safety Essentials: Understanding Risk Factors	5.00 (0.00)	4.82 ± 0.42	96.40%	1	1	5 (0.00)	5.00 ± 0.00	100%	1	1
		Understanding Team Dynamics & Collaboration*	5.00 (0.00)	4.91 ± 0.32	98.20%	1	1	5 (0.00)	5.00 ± 0.00	100%	1	1
		Assigning a Mentor & Building Professional Relationships	5.00 (0.00)	5.00 ± 0.00	100.00%	1	1	5 (0.00)	5.00 ± 0.00	100%	1	1
Module 2	Guidance & Mentorship	Setting Professional Goals With the Mentor	5.00 (0.00)	4.91 ± 0.32	98.20%	1	1	5 (0.00)	5.00 ± 0.00	100%	1	1
		Introduction to Key Team Members	5.00 (0.00)	4.91 ± 0.32	98.20%	1	1	5 (0.00)	5.00 ± 0.00	100%	1	1
		Safe Communication in Patient Handoffs	5.00 (0.00)	4.82 ± 0.42	96.40%	1	1	5 (0.00)	5.00 ± 0.00	100%	1	1
Day 2	Continuation	Introduction to Incident Reporting Systems	5.00 (0.00)	4.82 ± 0.42	96.40%	1	1	5 (0.00)	5.00 ± 0.00	100%	1	1
		Operational Challenges & Evidence-Based Solutions	5.00 (1.00)	4.73 ± 0.48	94.60%	1	1	5 (0.00)	5.00 ± 0.00	100%	1	1
		Access to Resources & Tools (Evidence-Based)	5.00 (0.00)	4.82 ± 0.42	96.40%	1	1	5 (0.00)	5.00 ± 0.00	100%	1	1
Module 3	Continuous Support	Stress Management Strategies	5.00 (1.00)	4.73 ± 0.48	94.60%	1	1	5 (0.00)	5.00 ± 0.00	100%	1	1
		Identifying & Preventing Patient Safety Risks	5.00 (0.00)	4.82 ± 0.42	96.40%	1	1	5 (0.00)	5.00 ± 0.00	100%	1	1
		Navigating Ethical Dilemmas in Daily Practice*	5.00 (0.00)	4.82 ± 0.42	96.40%	1	1	5 (0.00)	5.00 ± 0.00	100%	1	1
		Medication Safety and Administration	5.00 (0.00)	4.91 ± 0.32	98.20%	1	1	5 (0.00)	5.00 ± 0.00	100%	1	1
		Infection Prevention and Control	5.00 (0.00)	4.91 ± 0.32	98.20%	1	1	5 (0.00)	5.00 ± 0.00	100%	1	1
		Monthly Continuing Education Sessions	5.00 (1.00)	4.73 ± 0.48	94.60%	1	1	5 (0.00)	5.00 ± 0.00	100%	1	1
Module 4	Feedback & Evaluation	Performance Evaluations & Goal Adjustment (Evidence-Based)	5.00 (1.00)	4.73 ± 0.48	94.60%	1	1	5 (0.00)	5.00 ± 0.00	100%	1	1
		Collecting Employee Feedback (Surveys & Interviews)	5.00 (0.00)	4.91 ± 0.32	98.20%	1	1	5 (0.00)	5.00 ± 0.00	100%	1	1
		Applying Feedback to Practice	5.00 (0.00)	4.82 ± 0.42	96.40%	1	1	5 (0.00)	5.00 ± 0.00	100%	1	1
		Team-building Activities & Cohesion	5.00 (0.00)	4.91 ± 0.32	98.20%	1	1	5 (0.00)	5.00 ± 0.00	100%	1	1
		Leadership & Professional Growth Opportunities	5.00 (0.00)	4.91 ± 0.32	98.20%	1	1	5 (0.00)	5.00 ± 0.00	100%	1	1
Module 5	Integration Into Work Environment	Professional Development Workshops	5.00 (0.00)	4.82 ± 0.42	96.40%	1	1	5 (0.00)	5.00 ± 0.00	100%	1	1
		Advanced Communication Strategies for Patient Advocacy*	5.00 (0.00)	4.82 ± 0.42	96.40%	1	1	5 (0.00)	5.00 ± 0.00	100%	1	1
		Long-Term Career Path Planning	5.00 (0.00)	4.82 ± 0.42	96.40%	1	1	5 (0.00)	5.00 ± 0.00	100%	1	1
		Improving Patient Safety Outcomes Through Collaboration	5.00 (0.00)	4.82 ± 0.42	96.40%	1	1	5 (0.00)	5.00 ± 0.00	100%	1	1
Overall Summary Response Rate = 100%			5 (0.00)	4.83 ± 0.53	96.71%	1	1	5 (0.00)	5.00 ± 0.00	100%	1	1

Final validated high-reliability program

Based on the Delphi consensus process, the HRP was finalized as a comprehensive, structured intervention comprising five core domains: Orientation, Guidance and Mentorship, Continuous Support, Feedback and Evaluation, and Integration into the Work Environment

Each domain includes clearly defined components designed to address key aspects of the nursing work environment and promote patient safety. The final validated structure of the HRP is presented in Table 6.

**Table 6:** Finalized Educational Module Program

Module Number	Module Title	Session Title	Duration: 4 hours 30 minutes	Teaching and Learning Methods
Day 1	General Orientation	Welcome Session: Overview of the Organization	45 minutes	Video Lectures: These consist of content lectures on topics related to the nursing work environment, patient safety, communication, and clinical decision-making. Readings: Summaries and evidence-based readings on the fundamental concepts, issues, and best practices in patient safety. Discussion Forums: Facilitated dialogue that allows participants to share insights, reflect upon their work, and discuss strategies for improving patient safety, collaboration, and advocacy skills.
		Facility Tour & Team Introduction		
		Organizational Policies & Culture		
Module 1	Orientation	Health & Safety Training (Evidence-Based)	45 minutes	
		Role Expectations & Responsibilities		
		Patient Safety Essentials: Understanding Risk Factors		
		Understanding Team Dynamics & Collaboration		
		Assigning a Mentor & Building Professional Relationships		
Module 2	Guidance & Mentorship	Setting Professional Goals With the Mentor	45 minutes	
		Introduction to Key Team Members		
		Safe Communication in Patient Handoffs		
Day 2	Continuation	Introduction to Incident Reporting Systems	45 minutes	
		Operational Challenges & Evidence-Based Solutions		
		Access to Resources & Tools (Evidence-Based)		
Module 3	Continuous Support	Stress Management Strategies	45 minutes	
		Identifying & Preventing Patient Safety Risks		
		Navigating Ethical Dilemmas in Daily Practice		
		Medication Safety and Administration		
		Infection Prevention and Control		
		Monthly Continuing Education Sessions		
Module 4	Feedback & Evaluation	Performance Evaluations & Goal Adjustment (Evidence-Based)	45 minutes	
		Collecting Employee Feedback (Surveys & Interviews)		
		Applying Feedback to Practice		
		Team-building Activities & Cohesion		
		Leadership & Professional Growth Opportunities		
Module 5	Integration Into Work Environment	Professional Development Workshops	45 minutes	
		Advanced Communication Strategies for Patient Advocacy		
		Long-Term Career Path Planning		
		Improving Patient Safety Outcomes Through Collaboration		

Content validity outcomes

The content validity analysis demonstrated excellent agreement among experts. All items achieved maximum scores for both validity measures (Table 7).

**Table 7:** Content Validity Assessment of the Instrument

Validity Measure	Score	Interpretation
Content Validity Index (CVI)	1.00	Indicates perfect agreement among experts regarding item relevance
Content Validity Ratio (CVR)	1.00	Reflects unanimous agreement on the essentiality of all items

These findings indicate unanimous agreement among the expert panel regarding the relevance and essentiality of all HRP components. The results exceed recommended thresholds for content validity, confirming that the program is robust, comprehensive, and suitable for implementation in clinical settings.

Overall, the findings demonstrate that the comprehensive HRP underwent a rigorous multi-round Delphi validation process and achieved full expert consensus, establishing its validity as an evidence-based intervention for improving the nursing work environment and patient safety.

## Discussion

This study developed and rigorously validated a comprehensive HRP designed to improve the nursing work environment and enhance patient safety through a structured, multidimensional intervention. The attainment of unanimous expert consensus (CVI = 1.00; CVR = 1.00) indicates strong agreement regarding the relevance and essentiality of program components, while also reflecting convergence in expert perspectives on key determinants of patient safety within nursing practice. While such complete agreement reflects conceptual clarity and alignment with contemporary patient safety priorities, it also warrants cautious interpretation. Delphi studies achieving perfect consensus may reflect shared professional paradigms or contextual similarities among experts, particularly when panel sizes are relatively small, potentially limiting the diversity of perspectives among experts. Nonetheless, the iterative refinement process and consistent stability across rounds strengthen confidence in the content validity and coherence of the HRP.

A central contribution of this study lies in its transition from predominantly observational evidence toward the development of an actionable, evidence-based intervention. Existing literature has consistently demonstrated that the nursing work environment significantly influences patient safety outcomes, with factors such as staffing adequacy, leadership support, communication, and access to resources playing critical roles [14,7,8]. However, most prior studies have relied on cross-sectional or correlational designs, limiting their capacity to inform practical interventions [8,14]. By operationalizing these determinants into a structured program, the present study advances the field toward translational application, addressing a well-documented gap between evidence generation and implementation.

Importantly, the HRP’s five core domains-orientation, guidance and mentorship, continuous support, feedback and evaluation, and integration into the work environment-collectively reflect a systems-oriented approach to patient safety. The orientation domain establishes foundational knowledge through a structured introduction to organizational policies, safety protocols, and role expectations. This component directly addresses role ambiguity and inadequate onboarding, which have been associated with increased clinical errors and reduced adherence to safety practices [6]. By incorporating patient safety fundamentals early in professional integration, the program aligns with evidence suggesting that early integration into structured clinical environments supports adherence to safety practices and improves role clarity [5,6].

The guidance and mentorship domain represents a critical mechanism for translating knowledge into practice. Organizational support mechanisms, including structured mentorship and professional guidance, have been associated with improved staff engagement, reduced burnout, and enhanced teamwork in healthcare settings [4]. Within the HRP, structured mentorship, goal setting, and communication training, particularly in handoffs and incident reporting, address well-documented sources of preventable harm, including communication failures and underreporting of adverse events [9,10]. The inclusion of these elements reflects high reliability principles, particularly preoccupation with failure and sensitivity to operations, by encouraging continuous vigilance and open communication.

The continuous support domain further strengthens the program by embedding ongoing education and skill reinforcement into routine practice. Components such as medication safety, infection prevention, and risk identification directly target common sources of adverse events in healthcare settings [14,7]. Additionally, the inclusion of stress management strategies acknowledges the impact of workload and burnout on clinical performance. This aligns with evidence indicating that occupational stress negatively affects decision-making, increases error rates, and compromises patient safety [18]. By addressing both technical competencies and psychosocial factors, this domain reflects a comprehensive understanding of safety as a multifactorial construct.

The feedback and evaluation domain introduces structured mechanisms for performance monitoring, continuous improvement, and organizational learning. Feedback systems and performance monitoring are important components of clinical governance that support accountability and reinforce adherence to safety standards [5]. Moreover, the incorporation of employee feedback mechanisms promotes a non-punitive safety culture, which is essential for encouraging error reporting and organizational transparency [10]. The addition of leadership development and team-building activities further supports the creation of a collaborative environment, consistent with evidence linking effective leadership to improved patient outcomes and workforce engagement [13].

Finally, the integration into the work environment domain ensures the sustainability and institutionalization of learned practices. By focusing on professional development, interdisciplinary collaboration, and long-term career planning, this domain facilitates the embedding of safety behaviors into everyday clinical workflows. This approach is consistent with Systems Theory, which emphasizes the interdependence of organizational components in shaping outcomes [21]. It also aligns with high-reliability organization principles, particularly commitment to resilience and continuous learning, by promoting adaptability and sustained performance improvement in complex healthcare environments.

From a theoretical perspective, the HRP is strongly grounded in the Job Demands-Resources (JD-R) model and Systems Theory. The JD-R model provides a useful framework for understanding how the program’s components function by introducing supportive work environment factors, such as training, leadership support, and communication, that enhance staff performance and mitigate workplace challenges [6]. Simultaneously, Systems Theory underscores the interconnected nature of the HRP domains, highlighting that improvements in patient safety are not attributable to isolated interventions but rather to coordinated changes across multiple organizational dimensions [21]. This theoretical integration strengthens the conceptual validity of the program and supports its applicability in complex healthcare settings.

The findings also have significant implications for clinical practice, healthcare management, and policy. The modular structure of the HRP allows for flexible implementation within existing organizational frameworks, such as nurse orientation programs, continuing professional development initiatives, and quality improvement strategies. This is particularly relevant for secondary healthcare settings, where limitations in staffing, training resources, and organizational support have been shown to negatively affect patient safety outcomes [15,16]. By providing a scalable, adaptable framework, the HRP offers a practical solution for enhancing patient safety across diverse contexts. Furthermore, the program aligns with broader patient safety priorities, including the promotion of safety culture, workforce development, and error reduction, making it relevant for policy-level integration.

Despite these strengths, several limitations should be considered. The relatively small sample size of the expert panel, although consistent with Delphi methodology, may limit the generalizability of the findings. Additionally, the potential for consensus bias cannot be excluded, as experts may share similar professional backgrounds or contextual experiences. The study also focused exclusively on content validation, without assessing the effectiveness of the HRP in real-world settings. As such, the practical impact of the program on patient safety outcomes remains to be empirically tested. Future research should prioritize the implementation and evaluation of the HRP using robust study designs, such as quasi-experimental or cluster randomized trials. Longitudinal studies are also needed to assess the sustainability of the program’s effects over time. Additionally, further research should explore the adaptation of the HRP across different healthcare contexts, including primary care and tertiary institutions, to enhance its generalizability and scalability. In summary, this study provides a theoretically grounded and empirically validated framework that translates established patient safety principles into a structured, implementable intervention. By integrating organizational, educational, and behavioral components, the HRP offers a comprehensive approach to improving the nursing work environment and advancing patient safety.

## Conclusions

This study provides a rigorously developed and expert-validated HRP that translates established patient safety principles into a structured, implementable framework for nursing practice. Beyond confirming content validity, the findings highlight the feasibility of integrating high-reliability principles into routine clinical workflows through targeted interventions addressing communication, mentorship, and organizational support. Given the increasing global emphasis on patient safety and healthcare quality, the HRP offers a scalable model for strengthening safety culture, particularly in resource-constrained settings. Future research should focus on empirical implementation and outcome evaluation to determine its effectiveness in reducing adverse events and improving clinical performance.
